# PPAR-Delta Agonist Therapies Did Not Rescue Hallmark Disease Phenotypes in Two Sets of Preclinical Trials in ALS TDP-43 and C9orf72 Model Mice

**DOI:** 10.3390/ijms27041820

**Published:** 2026-02-13

**Authors:** David T. Luong, Chenchen Niu, Eunice Kim, Nolan Tanji, Ivy Duong, Brandon Galero, Yong-Jie Zhang, Craig L. Bennett, Albert R. La Spada

**Affiliations:** 1 Department of Pathology and Laboratory Medicine, University of California, Irvine, CA 92697, USA; 2 Department of Neuroscience, Mayo Clinic, Jacksonville, FL 32224, USA; 3 UCI Center for Neurotherapeutics, University of California, Irvine, CA 92697, USA; 4 Department of Neurology, University of California, Irvine, CA 92697, USA; 5 Department of Biological Chemistry, University of California, Irvine, CA 92697, USA; 6 Department of Neurobiology and Behavior, University of California, Irvine, CA 92697, USA

**Keywords:** amyotrophic lateral sclerosis, C9orf72, AAV-149R, TDP-43, PPARδ, KD3010, T3D-959, dipeptide repeat, neurofilament light chain

## Abstract

Peroxisome-proliferator–activated receptor delta (PPARδ) regulates metabolic, mitochondrial, and inflammatory pathways implicated in neurodegeneration, making it an attractive therapeutic target for amyotrophic lateral sclerosis (ALS). In this study, we evaluated two PPARδ agonists, KD3010 and T3D-959, in two established ALS/FTD mouse models: an AAV-mediated C9orf72 G4C2-repeat expansion model (C9-149R) and the TDP-43^Q331K^ transgenic model. Drug treatment was initiated prior to the emergence of key disease features and continued for 9–10 months. Comprehensive behavioral, neuropathological, and biomarker analyses revealed marked differences between the two models. C9-149R mice exhibited reduced body weight and subtle behavioral alterations without robust motor deficits, whereas TDP-43^Q331K^ mice developed pronounced, progressive motor and cognitive impairments accompanied by a ~7-fold elevation in plasma neurofilament light chain (NfL). Despite effective target engagement—particularly for T3D-959—neither PPARδ agonist improved motor performance, cognitive behavior, neuroanatomical measures, plasma NfL levels, or disease-associated molecular phenotypes in either model. Prolonged KD3010 treatment resulted in loss of target engagement, consistent with drug tolerance, while T3D-959 sustained PPARδ activation without therapeutic benefit. Together, these findings demonstrate that PPARδ agonism is insufficient to modify disease progression in these ALS/FTD mouse models and underscore the importance of publishing well-powered negative preclinical studies to refine therapeutic strategies for ALS.

## 1. Introduction

### 1.1. The Need for New ALS Therapeutics

Amyotrophic lateral sclerosis (ALS) is a rapidly progressive neurodegenerative disorder characterized by the selective degeneration of upper and lower motor neurons in the motor cortex, brainstem, and spinal cord [1]. Progressive motor neuron loss leads to muscle weakness, spasticity, atrophy, and ultimately death, most commonly due to respiratory failure within 3–5 years of diagnosis. Despite being the most common neurodegenerative disease of midlife, ALS remains largely untreatable. Currently approved FDA therapies for ALS provide, at best, modest clinical benefit. Riluzole and edaravone, the longest-standing approved treatments, primarily target mechanisms related to neuronal hyperexcitability and oxidative stress, respectively [1,2,3]. More recently approved therapies, including sodium phenylbutyrate/taurursodiol and dextromethorphan/quinidine, address additional cellular stress pathways but similarly confer only limited survival or functional benefit [4,5,6]. Collectively, these therapies extend survival by only a few months, underscoring the urgent need for more effective disease-modifying treatments. The persistent failure of ALS clinical trials reflects several interrelated challenges. First, available ALS animal models incompletely recapitulate the complexity and heterogeneity of human disease. Second, preclinical studies frequently emphasize positive outcomes while underreporting negative or null results, limiting objective assessment of therapeutic potential. Third, compounds that demonstrate biological activity or target engagement may nevertheless fail to meaningfully modify disease-relevant phenotypes. Addressing these issues requires rigorously designed preclinical studies that evaluate both therapeutic promise and limitations across multiple disease models. In this study, we employed two well-characterized ALS/FTD mouse models—the AAV-mediated C9orf72 G4C2-repeat expansion model and the TDP-43^Q331K^ transgenic model—to test whether pharmacologic activation of peroxisome proliferator–activated receptor delta (PPARδ) can ameliorate disease-associated phenotypes. By applying long-term treatment with two distinct PPARδ agonists and conducting comprehensive behavioral, pathological, and biomarker analyses, we aimed to critically assess the therapeutic potential of PPARδ activation in ALS. Given the central involvement of metabolic stress, mitochondrial dysfunction, and neuroinflammation in ALS pathogenesis, nuclear receptor pathways that regulate these processes represent attractive therapeutic targets. Peroxisome proliferator–activated receptor delta (PPARδ) is highly expressed in neurons and has been shown to support mitochondrial function, energy homeostasis, and inflammatory regulation in the nervous system. These properties provided a rationale for evaluating pharmacologic activation of PPARδ as a potential disease-modifying strategy in ALS, as explored in detail below.

### 1.2. The C9ORF72 Gene Dipeptide Repeat Mouse Model Based on AAV Expression of 149 Repeats

The *C9ORF72* gene is a major contributor to ALS, with its G4C2-repeat expansion mutation responsible for up to 50% of familial ALS (FALS) and 5–10% of sporadic ALS (SALS) cases [7]. Given the substantial clinical and pathological overlap between ALS and frontotemporal dementia (FTD), this same mutation also accounts for approximately one-third of familial FTD cases [8]. Three primary mechanisms of *C9ORF72*-mediated toxicity have been proposed: (1) RNA gain of function, (2) loss of function, and (3) toxic dipeptide repeat (DPR) production through repeat-associated non-AUG (RAN) translation [9]. Notably, arginine-rich DPRs, such as poly-GR and poly-PR, disrupt membraneless organelles, including the nucleolus and nuclear pore complexes, leading to impaired nucleocytoplasmic transport and cellular dysfunction [10,11]. Several mouse models have been developed to study *C9ORF72*-associated ALS/FTD. Transgenic mice expressing ~500 repeats initially showed disease-associated features [12], but subsequent independent studies failed to identify consistent ALS-like phenotypes [13]. A bacterial artificial chromosome (BAC) model expressing 450 repeats produced RNA foci and DPRs but lacked significant neurodegeneration or glial activation [14]. Together, these findings underscored the challenges of modeling *C9ORF72*-mediated neurodegeneration in mice. A major turning point for modeling *C9ORF72* ALS came with the development of an AAV-based model expressing 149 G4C2 repeats, which recapitulates key features of clinical disease, including neurodegeneration, cognitive and motor deficits, gliosis, RNA foci, and DPR accumulation by six months of age, accompanied by hallmark ALS/FTD pathology [15]. This model was therefore used in the preclinical studies we conducted, with phenotype-characterizing assays akin to those previously reported being employed.

### 1.3. The ALS TDP-43^Q331K^ Transgenic Mouse

The identification of TDP-43 nuclear clearance and cytoplasmic aggregation as hallmark pathologies in ALS represented a major advance in understanding disease mechanisms. Such pathology is present in nearly all forms of ALS, including both sporadic and familial cases, with the notable exception of SOD1-associated ALS [16]. This observation motivated the development of mouse models to study TDP-43-mediated neurodegeneration. A transgenic model was generated in which either wild-type TDP-43 or the Q331K mutant form (TDP-43^wt^, TDP-43^Q331K^) is expressed under the control of the mouse prion promoter (MoPrP). In this model, combined transgene and endogenous TDP-43 mRNA levels are approximately three-fold higher than are those in non-transgenic mice, and the MoPrP drives robust expression throughout the neuromuscular axis [17]. The TDP-43^Q331K^ transgenic line develops age-dependent axonal degeneration and motor neuron loss [18]. Interestingly, lower motor neuron degeneration in this model occurs in the absence of overt TDP-43 nuclear clearance or cytoplasmic aggregation but is nonetheless associated with mRNA splicing defects linked to ALS [18]. This feature distinguishes the TDP-43^Q331K^ mouse from aggregation-centric models and highlights its relevance for studying TDP-43-dependent RNA dysregulation. As one of the most well-characterized mouse models capturing TDP-43-mediated molecular and functional abnormalities, the TDP-43^Q331K^ transgenic line was also selected for testing PPARδ agonist therapy in the present study.

### 1.4. PPARδ Activation Supports Neuroprotective Cellular and Molecular Pathways

PPARδ is a nuclear hormone receptor that regulates diverse biological processes, including lipid metabolism, mitochondrial function, and inflammatory signaling. We previously reported that PPARδ is highly expressed in neurons and that its targeted inhibition leads to progressive neurodegeneration in mice [19]. In prior studies, treatment of Huntington’s disease mouse models with the PPARδ agonist KD3010 (30 mg/kg intraperitoneal injection, administered Monday, Wednesday, and Friday) markedly prevented neurodegeneration and extended lifespan [19]. In addition, cortical neurons isolated from BAC-HD mice exhibited significant reductions in oxygen consumption rate (OCR), a deficit that could be reversed by KD3010 treatment in vitro [20]. KD3010 treatment was also associated with improvements in protein quality control and mitochondrial fragmentation in these neurons [20]. Collectively, these findings suggested that pharmacologic activation of PPARδ may support neuronal metabolic function and viability. T3D-959 (T3D Therapeutics) is a dual PPARδ/γ agonist, with substantially greater potency for PPARδ (human EC50 = 19 nM) than for PPARγ (human EC50 = 297 nM). In a pilot study using a streptozotocin-induced rat model of Alzheimer’s disease, treatment with T3D-959 (0.3–3.0 mg/kg/day for 28 days) improved motor performance, reduced neuroinflammation, enhanced brain insulin/IGF-1 signaling, and preserved cortical neurons [21]. These findings were consistent with other published reports [21,22]. Together with previously published T3D-959 studies [21,22,23], these data supported evaluation of T3D-959 in neurodegenerative disease models. Notably, T3D-959 has advanced through an ascending-dose clinical trial in human volunteers without serious adverse events (DB959-102). Notably, T3D-959 has advanced through early-phase clinical testing in Alzheimer’s disease, where it demonstrated acceptable safety and pharmacodynamic target engagement in a randomized phase 1b/2a trial [24]. In the present study, T3D-959 was administered at 50 mg/kg by intraperitoneal injection on a Monday–Wednesday–Friday schedule.

The selection of these two ALS/FTD mouse models was intentional and central to the experimental design. The AAV-mediated C9-149R model captures core molecular and pathological features of C9orf72-associated ALS/FTD, including dipeptide repeat production and TDP-43 pathology, but exhibits relatively modest and variable motor phenotypes. In contrast, the TDP-43Q^331K^ transgenic model develops robust, progressive motor and behavioral deficits driven by TDP-43-dependent RNA dysregulation. Together, these models provide complementary disease contexts in which to evaluate therapeutic efficacy, allowing assessment of PPARδ agonism under both mild and aggressive disease conditions. This dual-model approach was designed to distinguish failure due to insufficient disease burden from failure despite substantial and progressive pathology, thereby strengthening the interpretation of negative findings.

Collectively, prior studies indicate that PPARδ agonists can modulate metabolic, mitochondrial, and inflammatory pathways relevant to neurodegeneration, supporting their evaluation in ALS/FTD mouse models. Based on these findings, we assessed the therapeutic potential of PPARδ activation in two well-characterized models: the AAV-mediated C9-149R ALS/FTD model and the TDP-43^Q331K^ transgenic ALS model. In the C9-AAV model, mice were treated with KD3010 or T3D-959 beginning at three months of age and continuing until twelve months of age. In the TDP-43^Q331K^ model, treatment with KD3010 or T3D-959 began at two months of age and continued until twelve months of age. Thus, C9-AAV mice received approximately nine months of treatment, whereas TDP-43^Q331K^ mice received approximately ten months of treatment.

## 2. Results

We found that significant phenotypic differences exist between the two ALS/FTD mouse models studied here. The therapeutic drug treatments applied to each were very similar, with a minor difference in initiation time. The C9-AAV repeat-expansion mice were on drug therapy for 9 months and the TDP-43^Q331K^ mice were treated for 10 months. C9-2R mice served as AAV-injected control animals expressing two G4C2 repeats, with viral delivery and transgene expression independent of repeat expansion length being controlled for, whereas C9-149R mice expressed pathogenic expanded repeats.

### 2.1. Body Weight Differences in AAV C9-149R and TDP-43Q331K Mice

**Figure 1:**
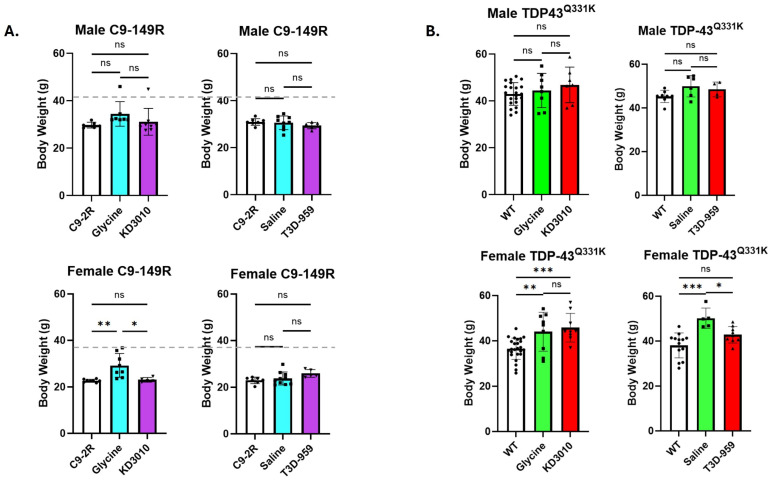
**Body weight at one year of age, with thrice weekly drug treatment for 9 months.** Final body weights were recorded at one year of age for each primary study cohort. Individual weights are represented as solid black circles (control), squares (vehicle-treated), or triangles (drug-treated). (**A**) For the C9-149R model, average body weights are given for C9-2R (open column) and C9-149R (blue column) mice treated with KD3010 (purple column). No reduction in body weight was observed except for females with the glycine vehicle control versus the C9-2R group. This likely reflects a statistical anomaly as this was not observed for the saline control. The mean weight for male and female WT mice is indicated by a gray dashed line. (**B**) For the TDP-43^Q331K^ model, average weights are shown as WT, open column; vehicle-treated (green column); and T3D-959-treated (red column). Female mice exhibited a modest but consistent weight gain compared to WT controls. Treatment with T3D-959, but not with KD3010, reduced the weight gain of females linked with TDP-43^Q331K^ expression. Within-group comparisons were analyzed using one-way ANOVA with Tukey’s post hoc test. Statistical significance is denoted as follows: *p* < 0.05 (*); *p* < 0.01 (**); and *p* < 0.001 (***). Error bars indicate ±SD.

Body weight was recorded for all cohorts at study completion (one year) (Figure 1), and at weekly intervals for the vehicle and drug-treatment groups (Supplemental Figure S1). For the C9-AAV model, there was one example in female mice where weight gain was significant at one year for the C9-149R glycine-treated group. However, this was not corroborated in the C9-149R saline-treated group (Figure 1A, lower panel). This finding might best be described as an anomaly, but the increase in female C9-149R glycine body weight was also apparent in the weekly testing (Supplemental Figure S1A, lower panel). For the TDP-43^Q331K^ trial, we observed a significant weight gain in both female cohorts over-expressing the TDP-43 mutation and receiving vehicle treatment (glycine and saline) (Figure 1B, lower panel). Importantly, only T3D-959 drug treatment significantly reduced body weight toward WT levels. KD3010 treatment did not produce a comparable reduction in body weight.

Interestingly, each of the AAV-injected mice (C9-2R and C9-149R) appears to have reduced body weight relative to WT mice. Given the absence of significant differences between the two C9-2R cohorts, data were combined for analysis (*n* = 14) and compared to the WT cohorts from the KD3010 study (*n* = 22), yielding mean weights of 30.5 ± 1.5 g and 43.0 ± 4.7 g, respectively. Similarly, by combining the two C9-2R female cohorts (*n* = 15), we have a mean body weight of just 22.8 ± 1.0 g and 36.6 ± 5.2 g for WT female mice (*n* = 25) (Figure 1) respectively. These comparisons between C9-2R and WT body weight were highly significant for male and female mice respectively.

### 2.2. Motor Performance Testing via Rotarod and Grip Strength

Motor performance was assessed using rotarod and grip strength testing. Because of minor differences in treatment initiation, we performed rotarod testing at 3, 6, 9, and 12 months of age in the C9-AAV mice and at 2, 4, 6, 8, 10, and 12 months of age in the TDP-43^Q331K^ trial. The same schedule was used for a grip strength test performed on all four legs.

**Figure 2:**
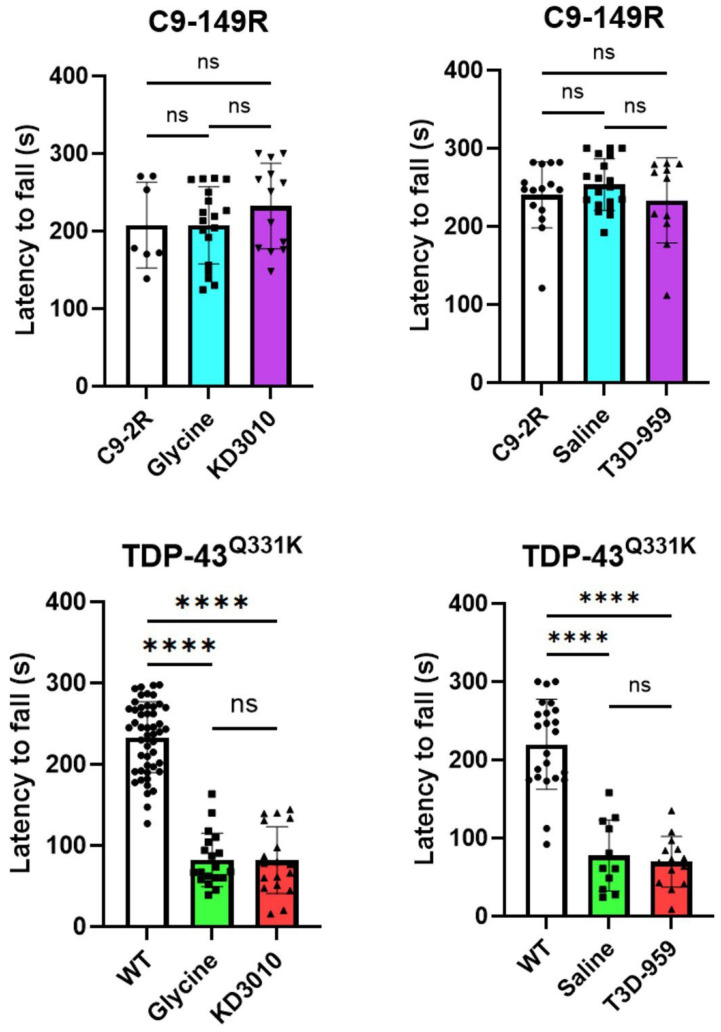
**Rotarod assessment of strength stamina and coordination.** At one year of age, mice were assessed for time to fall on an accelerating rotarod treadmill. The average latency to fall is represented by solid black circles (control), squares (vehicle-treated), or triangles (drug-treated). In the C9-149R model, average results are shown as C9-2R (open column), vehicle-treated (blue column), and drug-treated (purple column). There was no significant difference in performance between vehicle-treated groups (glycine or saline) and C9-2R controls (upper panel). For the TDP-43^Q331K^ model, average results are shown as WT (open column), vehicle-treated (green column), and drug-treated (red column). Motor performance was significantly reduced in all TDP-43^Q331K^ cohorts compared to WT controls (*p* < 0.0001, ****, one-way ANOVA with Tukey’s post hoc test). Error bars represent ±SD.

**Figure 3:**
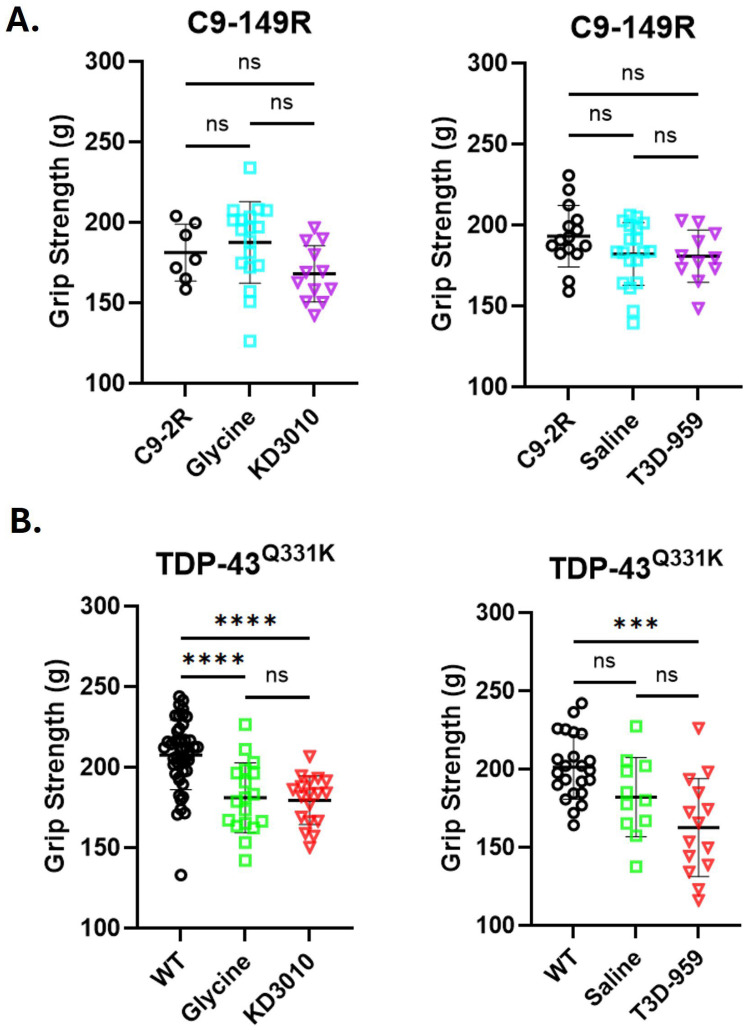
**Grip strength assessment.** At one year of age, mice were evaluated for all-limb grip strength using a grip meter (Bioseb #BIO-GS3). (**A**) The highest force recorded across trials for each mouse was averaged and displayed as open symbols: black circles, light blue squares, and purple triangles (n ≥ 7) for C9-2R, C9-149R vehicle, and C9-149R drug treatment groups, respectively. No significant grip strength difference was observed between the AAV trial cohorts. (**B**) In the TDP-43^Q331K^ trial, grip strength results for individual mice are shown as open black circles, light green squares, and red triangles (n ≥ 11) for WT, TDP-43^Q331K^ vehicle, and TDP-43^Q331K^ drug treatments, respectively. Significant grip strength deficits were observed in both vehicle-treated (glycine or saline) and drug-treated TDP-43^Q331K^ mice compared to WT controls (*p* < 0.001, ***; *p* < 0.0001, ****; one-way ANOVA with Tukey’s post hoc test). Error bars represent ±SD.

We found that the C9-149R mice did not show any rotarod deficit at 12 months of age (Figure 2, upper panel), and no deficit was observed at the 3-, 6-, and 9-month time points versus the C9-2R control mice (Supplemental Figure S2A). Similarly, no strength deficit was observed in these mice at one year (Figure 3, upper panel) or at 3, 6, 9, and 12 months (Supplemental Figure S3A). Accordingly, no opportunity existed for either KD3010 or T3D-959 to rescue a strength deficit in this model.

For the TDP-43^Q331K^ trial, there was a significant drop in rotarod performance versus WT mice for all four independent Q331K cohorts (Figure 2, lower panel). A statistically significant deficit was observed as early as 4 months, which became progressively worse over time (Supplemental Figure S2B). A significant grip strength deficit was detected at the study end for the glycine/KD3010 cohorts (Figure 3, lower panel), and a milder deficit was detected at 10 months of age in the same cohorts (Supplemental Figure S3B, upper panel). Statistical significance was reached in the saline/T3D-959 cohort at 8 months only (Supplemental Figure S3B, upper panel). This suggests that strength deficits are less pronounced than are stamina/coordination deficits in the TDP-43^Q331K^ model. Overall, the strong motor deficit demonstrated in the TDP-43^Q331K^ mice was not rescued or mitigated by KD3010 or T3D-959 treatment.

### 2.3. Behavioral Phenotype Testing

#### 2.3.1. Composite Neurological Assessment

**Figure 4:**
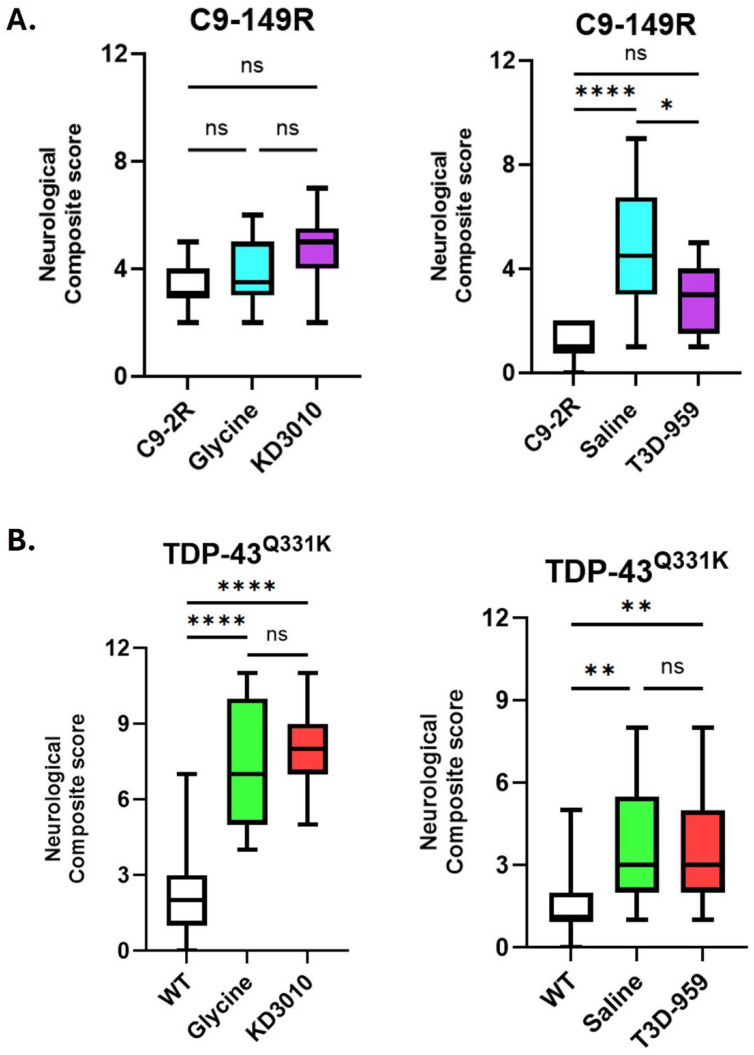
**Neurological assessment score (NCS).** At one year of age, mice were evaluated for neurobehavioral abnormalities using a graded composite scale. The average composite score for each cohort is shown as a box and whiskers plot. (**A**) For the AAV trial, results for C9-2R are shown as open black boxes; for C9-149R vehicle control, as light blue boxes; and for the C9-149R drug treatment, as purple boxes (n ≥ 7, per cohort). One significant difference in NCS was observed for the saline cohort versus the C9-2R control. This likely represents an anomaly driven by an uncharacteristically low C9-2R control NCS score of 1.2, a finding that was not supported by testing at earlier time points (see supplemental data). (**B**) In the TDP-43^Q331K^ trial (n ≥ 8 per cohort), results for WT cohorts are shown as open black boxes; for the TDP-43^Q331K^ vehicle cohorts, as light green boxes; and for the TDP-43^Q331K^ drug treatments, as purple boxes. Significant NCS deficits were observed in both vehicle-treated (glycine or saline) and drug-treated TDP-43^Q331K^ mice compared to WT controls (*p* < 0.05 *, *p* < 0.01 **; *p* < 0.0001 ****; one-way ANOVA with Tukey’s post hoc test). Whiskers represent the maximum and minimum NCS.

A previously developed composite neurological assessment was used to evaluate disease severity in mouse models, including ataxia, Huntington’s disease, and spinobulbar muscular atrophy [25]. This assessment included hind-limb clasping, ledge test performance, gait abnormalities, and kyphosis, yielding a maximum combined score of 12 (0 = young normal; 12 = severe disease). Neurological composite scores (NCSs) were not statistically different between C9-149R glycine/KD3010 cohorts and C9-2R control mice at one year of age (Figure 4A, upper left panel). In contrast, C9-149R saline/T3D-959 cohorts exhibited significantly higher average NCSs compared to C9-2R controls at one year (Figure 4A, upper right panel). When assessed at 6 and 9 months of age, these same saline/T3D-959 cohorts demonstrated a consistent, modest elevation in NCSs relative to controls (Supplemental Figure S4A, lower panel). Together, these findings indicate that C9-149R mice exhibit mild and variable neurological deficits as measured by this assay. No improvement in NCSs was observed with the PPARδ agonist treatment in this model. All four TDP-43^Q331K^ cohorts (glycine/KD3010 and saline/T3D-959) exhibited significantly higher NCSs compared to WT controls (*p* < 0.01 or greater) (Figure 4B). Longitudinal assessment at 6, 9, and 12 months of age revealed a consistent progression of neurological deficits (Supplemental Figure S4B). Despite the robustness of this phenotype, no significant differences in NCS scores were observed between drug-treated and vehicle-treated TDP-43^Q331K^ mice at any time point.

#### 2.3.2. Contextual Fear Conditioning Test

**Figure 5:**
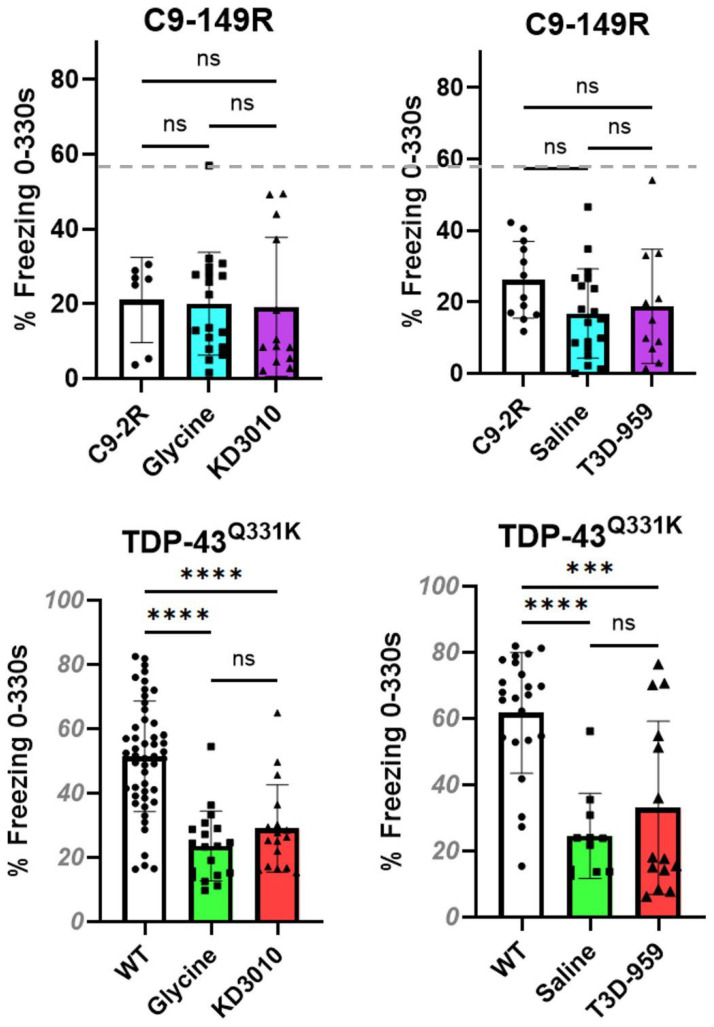
**Contextual fear conditioning (0–330 s).** Cognitive function was assessed by contextual fear conditioning to test for cognitive impairment, and individual results are shown as solid circles (control), squares (vehicle control), or triangles (drug-treated). Upper panel: At one year for the C9-149R model, the average percentage freezing time is shown for the C9-2R model (open columns), the C9-149R vehicle group (blue columns), and the C9-149R drug treatment group (purple columns). There was no significant difference between vehicle-treated or drug-treated cohorts and the C9-2R control. The mean percentage freezing time for WT mice is indicated by a gray dashed line. Lower panel: For the TDP-43^Q331K^ model, the results are presented as an open column–WT; green column–vehicle-treated; or purple column–drug-treated. In all cases, the Tg-mice showed a significantly reduced percentage freezing time compared with the WT control (*p* < 0.001, ***; *p* < 0.0001, ****); one-way ANOVA with Tukey’s post hoc test. Error bars represent ±SD. In the C9-AAV trial, the percentage freezing time ranged from just 16.8% to 26.2%, while for the TDP-43^Q331K^ trial, the range was much greater at 23.5% to 61.8%.

This assessment was undertaken with mice at one year of age, and we followed the contextual fear-conditioning methods previously used to analyze AAV C9-149R mice [15]. Focusing on the AAV-C9 model, we found no significant difference in freezing behavior between the two C9-149R vehicle cohorts and the two C9-149R drug-treatment cohorts compared with C9-2R controls (Figure 5, upper panel). Across all six AAV-C9 test cohorts, the average freezing time range was relatively tight, ranging from 16.8% to 26.2%. In contrast, results for the TDP-43^Q331K^ mice were markedly different. For the four TDP-43^Q331K^ transgenic cohorts (glycine, KD3010, saline, T3D-959), all displayed significantly reduced freezing behavior percentages compared with the WT control, consistent with impaired contextual fear conditioning (Figure 5, lower panel).

Similar to body weight being different between WT and C9-2R controls, we again found a difference in baseline percentage freezing times. Specifically, the two independent WT cohorts showed similarly high levels of freezing behavior (indicative of a healthy conditioning response) with average freezing percentages of 51.5% and 61.75%, while the two C9-2R cohorts averaged just 26.2% and 21.0%. By combining all C9-2R mice tested (*n =* 19) versus all WT mice from the KD3010 trial (*n* = 49), we detected a highly significant difference in this behavioral testing paradigm. To further examine freezing behavior, the day 2 analysis phase was subdivided to focus on the 120–330 s interval, which may emphasize sustained fear responses independent of initial contextual recall. Analysis of this interval yielded results largely consistent with those obtained using the full 330 s period. However, within the 120–330 s window, a modest but significant reduction in freezing behavior was observed in the C9-149R saline- and C9-149R T3D-959-treated cohorts relative to C9-2R controls (Supplemental Figure S5, upper right panel). As in prior behavioral assays, a robust and consistent phenotype was observed only in the TDP-43^Q331K^ model, with no evidence of rescue or amelioration by KD3010 or T3D-959 treatment.

#### 2.3.3. Open Field Test

**Figure 6:**
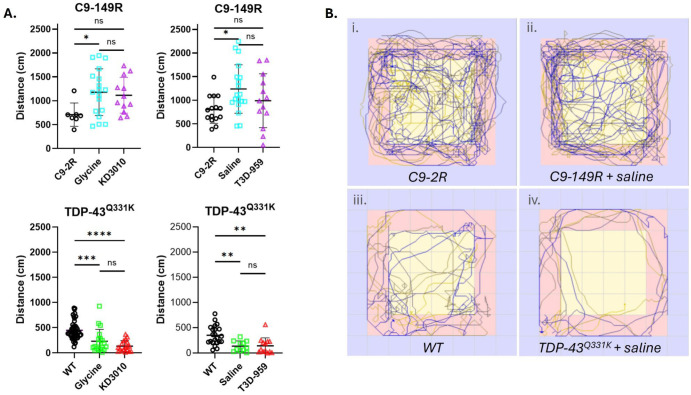
**Open field test.** The phenotypes in these two ALS/FTD models have opposite effects, with hyperactivity in the C9-AAV model and reduced overall movement in the TDP-43^Q331K^ mice. The total distance traveled in centimeters (cm) for each mouse tested is presented. (**A**). Upper panel: We employed the open-field assay to test for activity level, and hyperactivity is presented as an early phenotype in these C9-149R mice [15]. Within the C9-AAV trial, results are shown as open symbols: black circles, C9-2R; light-blue squares, vehicle-treated; and purple triangles, drug-treated. At the *p* < 0.05 significance level, the total distance traveled was increased in all C9-149R cohorts versus the C9-2R control. The mean distance traveled (cm) by WT mice is indicated by a gray dashed line. (**A**). Lower panel: In the TDP-43^Q331K^ trial, results are shown as open black circles, light green squares, and red triangles for WT, TDP-43^Q331K^ vehicle, and TDP-43^Q331K^ drug treatments, respectively. The total distance traveled was significantly reduced in both vehicle-treated and drug-treated cohorts. *p* < 0.05, *; *p* < 0.01, **; *p* < 0.001, ***; and *p* < 0.0001, **** one-way ANOVA with Tukey’s post hoc test. Error bars represent ±SD. (**B**): Open field tracking is shown for one representative male from each of the following cohorts: (**i**) C9-2R, (**ii**) C9-149R + saline, (**iii**) WT, and (**iv**) TDP-43Q331K + saline.

Open field testing was used to assess locomotor activity and exploratory behavior. In the AAV-C9 model, a modest increase in locomotor activity was detected in both C9-149R vehicle-treated cohorts compared with C9-2R control mice, consistent with previous reports for this model [15]. This difference reached statistical significance (Figure 6A, upper panel). In contrast, all four independent TDP-43^Q331K^ cohorts exhibited significantly reduced distance traveled relative to WT controls (Figure 6A, lower panel). Representative open field tracking plots from male mice are shown in Figure 6B for the following groups: (i) C9-2R, (ii) C9-149R + saline, (iii) WT, and (iv) TDP-43^Q331K^ + saline. The examples from WT and C9-2R mice illustrate pronounced baseline differences in locomotor behavior between these control cohorts, consistent with differences observed previously for body weight and contextual fear conditioning. Despite the presence of measurable behavioral phenotypes, neither KD3010 nor T3D-959 treatment altered open field performance in either model.

Consistent with findings in other behavioral assays, baseline open field performance differed between WT and C9-2R control mice. When all C9-2R mice tested (*n* = 24) were compared with all WT mice from the KD3010 trial (*n* = 49), there was a highly significant difference in distance traveled (772.19 ± 275.3 cm vs. 446.21 ± 183.1 cm; *p* = 1.27 × 10^−7^).

### 2.4. Neuroanatomy Assessment

#### ChAT-Positive Motor Neurons and NMJ Assessment

**Figure 7:**
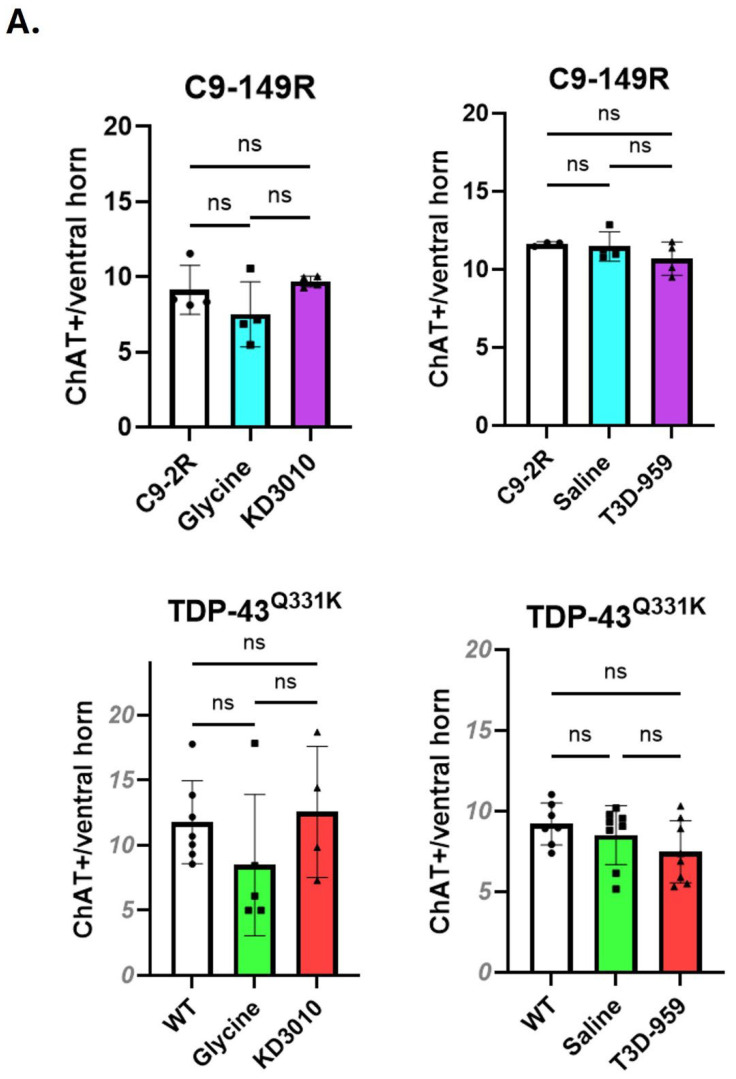

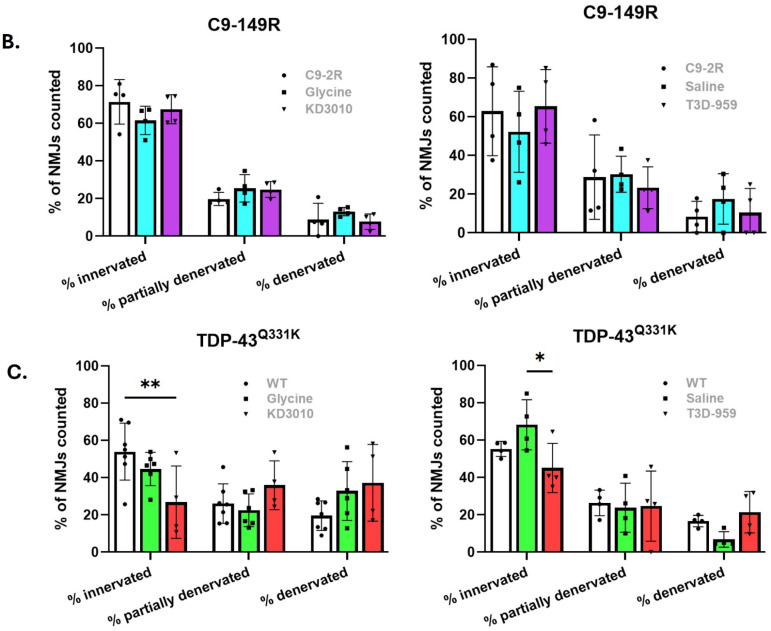
**ChAT+ motor neuron and neuromuscular junction (NMJ) number.** The average number of ChAT+ MNs per ventral horn of the spinal cord and characterization of gastrocnemius NMJs were assessed at one year of age for both mouse models. Average results for individual mice are indicated as solid circles (control), squares (vehicle-control), and triangles (drug-treated). (**A**) Upper panel. Results are shown as an open bar for C9-2R, a blue bar for C9-149R-vehicle, and a purple bar for C9-149R-drug treatment. In the C9-149R model, there was no significant difference between vehicle-treated or drug-treated cohorts and the C9-2R control. (**A**) Lower panel. For the TDP-43^Q331K^ model, the results are presented for an open bar for WT, a green bar for vehicle-treated, and a red column for drug-treated. No statistically significant drop in the number of ChAT+ motor neurons was found in any test cohort versus the WT control. (**B**,**C**) Neuromuscular junctions were also assessed for the C9-149R and TDP-43^Q331K^ mouse models, respectively. Using the same color pattern as above, the percentage of junctions is given for the percentage NMJs: innervated; partially denervated; and denervated. All intergroup comparisons were undertaken by one-way ANOVA with Tukey’s post hoc test (*p* < 0.05, *; *p* < 0.01, **). Error bars represent ±SD. We note that the only significant differences were between WT and TDP-43^Q331K^-KD3010 treated and between TDP-43^Q331K^-saline and TDP-43^Q331K^-T3D-959. These specific comparisons did not validate an ALS phenotype or drug-treatment rescue and were not considered further.

In the initial characterization of TDP-43^Q331K^ mice, a mild but statistically significant reduction in choline-acetyltransferase-positive (ChAT^+^) lower motor neurons was reported by 10 months of age [18]. ChAT^+^ motor neuron loss was not previously assessed in the AAV C9-149R model, and such loss was not anticipated based on the relatively mild motor phenotype described for this model [15]. Accordingly, ChAT^+^ motor neuron counts were evaluated in both models in the present study. More than 16 ventral horn sections per mouse were analyzed for ChAT^+^ motor neurons (*n* = 3 mice per group). At one year of age, no significant loss of ChAT^+^ motor neurons was detected in any of the four TDP-43^Q331K^ cohorts (Figure 7A, lower panel). Similarly, no significant reduction in ChAT^+^ motor neuron number was observed in any of the four AAV C9-149R cohorts (Figure 7A, upper panel).

Neuromuscular junctions (NMJs) were also assessed in both models, and the proportions of innervated, partially denervated, and fully denervated NMJs were quantified. No significant NMJ deficits were detected in C9-149R mice (Figure 7B). In the TDP-43^Q331K^ model, only two pairwise comparisons reached statistical significance (Figure 7C), neither of which involved comparisons between transgenic vehicle-treated mice and WT controls. In the absence of a consistent ChAT^+^ motor neuron or NMJ phenotype, meaningful assessment of KD3010 or T3D-959 treatment effects was not possible.

### 2.5. Neurofilament Light Chain (NfL); A Biomarker of Neuronal Death and Dysfunction

NfL is a widely used biomarker of neuronal injury and dysfunction. Although emerging biomarkers such as cardiac troponin T (cTnT) may offer increased specificity for ALS and FTD [26], plasma NfL remains the most commonly used biomarker for assessing neuronal injury in ALS animal models. While plasma NfL measurements were not included in the original characterization of TDP-43^Q331K^ mice, subsequent studies using the AAV C9-149R model demonstrated the utility of plasma NfL as a sensitive indicator of neuronal health [27].

**Figure 8:**
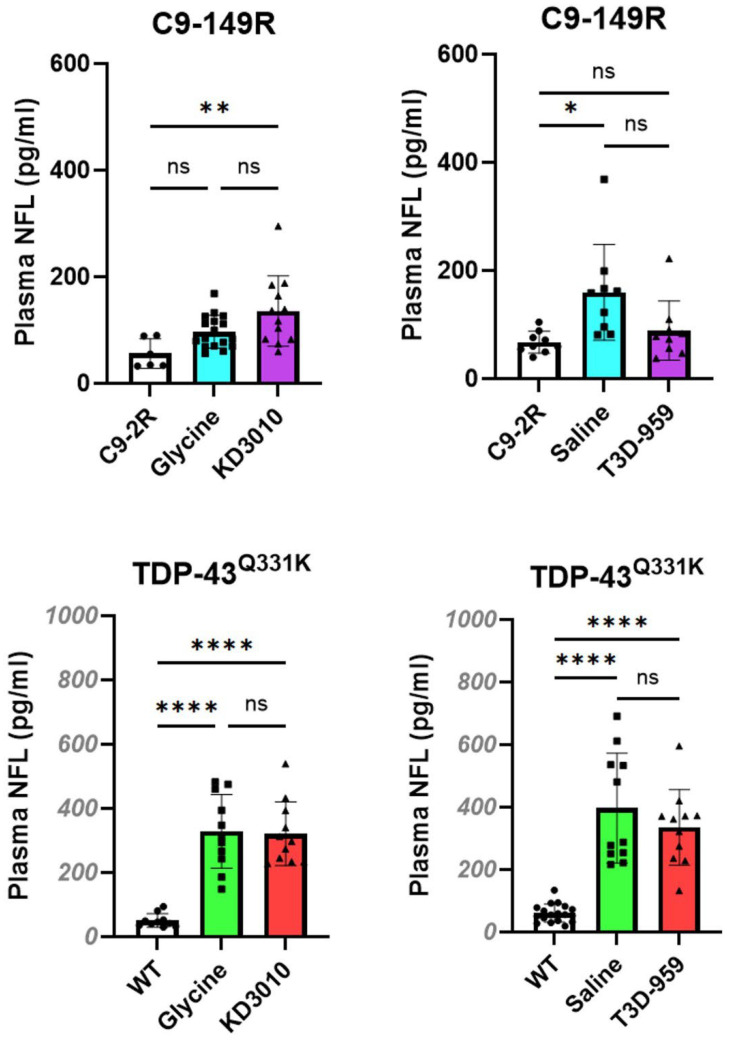
**Plasma neurofilament light chain (NfL) levels**. The NfL results for individual mice are indicated for control cohorts as solid circles, for ALS vehicle-control as squares, and for ALS drug-treated mice as triangles. The y-axis scale for the C9-AAV model is 0–600 pg/mL (upper panel) and 0–1000 pg/mL for the TDP-43^Q331K^ model (lower panel). Upper panel: In the C9-149R model, results are shown for C9-2R (open column), C9-149R-vehicle (blue column), and_ C9-149R-drug treatment (purple column). There was a significant elevation in plasma NfL for C9-149R KD3010-treated mice versus the C9-2R control. There was also a mild elevation for C9-149R/saline mice versus the C9-2R control. Lower panel: In the TDP-43^Q331K^ model, results are shown for WT (open column), glycine-vehicle (green column), and drug-treated (red column). There was a significant elevation in plasma NfL for all TDP-43^Q331K^ cohorts versus the WT control. *p* < 0.05, *; *p* < 0.01, **; and *p* < 0.0001, **** one-way ANOVA with Tukey’s post hoc test. Error bars represent ±SD.

All plasma NfL measurements in the present study were performed at one year of age. In the AAV C9-149R model, two of the four 149R cohorts exhibited significantly elevated plasma NfL levels relative to C9-2R controls. The C9-149R KD3010-treated cohort had mean NfL levels of 135.8 ± 65.9 pg/mL compared with 56.0 ± 27.5 pg/mL in C9-2R controls (*p* < 0.01), while the C9-149R saline-treated cohort showed mean NfL levels of 159.7 ± 88.7 pg/mL compared with 67.3 ± 20.3 pg/mL in controls (*p* < 0.05) (Figure 8, upper panel). In contrast, all four TDP-43^Q331K^ cohorts exhibited markedly elevated plasma NfL levels relative to WT controls (Figure 8, lower panel). Mean NfL levels in the glycine-control and KD3010-treated cohorts were 374.3 ± 206.9 pg/mL and 365.3 ± 168.0 pg/mL, respectively, compared with 50.8 ± 21.2 pg/mL in WT controls (ANOVA, *p* < 0.0001 for both comparisons). Similarly, saline-control and T3D-959–treated cohorts exhibited mean NfL levels of 375.6 ± 180.8 pg/mL and 396.8 ± 175.8 pg/mL, respectively, compared with 60.7 ± 28.8 pg/mL in WT controls (ANOVA, *p* < 0.0001 for both comparisons). Across both models, treatment with KD3010 or T3D-959 did not significantly reduce plasma NfL levels.

### 2.6. Validation of ALS/FTD Model Sub-Phenotypes and Drug Target Engagement

Key molecular and pathological features were assessed to validate disease-associated sub-phenotypes in both ALS/FTD mouse models. In the TDP-43^Q331K^ germline transgenic model, the presence of the mutation-associated cryptic exon was confirmed. In the AAV C9-149R model, expression of the poly-GP DPR, phosphorylated TDP-43 (pTDP-43), and C9-149R mRNA were detected, consistent with previously reported disease-associated molecular features.

#### 2.6.1. Validation of the TDP-43^Q331K^ Model

RNA was isolated from the cortex of one-year-old mice to assess cryptic exon splicing associated with TDP-43 dysfunction. Aberrant splicing of the target genes *Kcnip2* and *Eif4h*, which are known to be affected by TDP-43 pathology [18], was analyzed by PCR amplification of cDNA. Increased cryptic exon inclusion was detected in *Kcnip2* and *Eif4h* transcripts in the TDP-43^Q331K^ glycine- and KD3010-treated cohorts (Supplemental Figure S6). Comparable splicing alterations were also observed in saline- and T3D-959-treated cohorts and in spinal cord tissue. These findings confirm the presence of characteristic TDP-43-associated splicing defects in the TDP-43^Q331K^ model at one year of age.

#### 2.6.2. Validation of C9orf72 AAV-149R Model

The AAV-149R model has been reported to induce sense and antisense RNA foci, accumulation of DPRs, and endogenous pTDP-43 pathology [15]. To assess these disease-associated features at one year of age, C9-149R expression, poly(GP) DPR accumulation, and pTDP-43 pathology were examined. RT-PCR using primers flanking the G4C2 repeat demonstrated robust C9-149R expression in the cortex of all four C9-AAV 149R cohorts (Supplemental Figure S7A). Consistent expression was also detected in spinal cord tissue from both saline- and T3D-959-treated cohorts (Supplemental Figure S7A, right panel). Poly(GP), a hallmark DPR species generated through RAN translation of the G4C2 repeat, accumulated in the cortex at one year of age and was quantified in C9-2R-, C9-149R glycine, and C9-149R KD3010-treated cohorts (Supplemental Figure S7B,C). In addition, pTDP-43 accumulation was observed in the cortex of C9-149R mice at one year of age.

#### 2.6.3. Validation of KD3010 and T3D-959 Target Engagement

**Figure 9:**
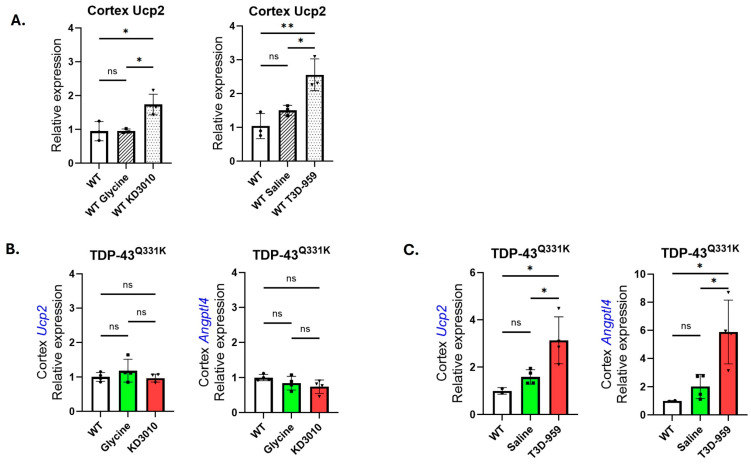
**PPARδ target gene engagement.** *Ucp2* and *Angptl4* are two key genes regulated by PPARδ agonism. Their expression levels were tested before the trial began and at the end of the study (12 months) using brain-isolated cDNA. This was performed to ensure the validity of the study. (**A**) The expression levels of *Ucp2* were tested in WT mice at 3 months of age following one week of our standard PPARδ treatment regime: (i) WT (open column); (ii) glycine or saline (striped); or (iii) KD3010 or T3D-959 (stippled). Importantly, treatment with PPARδ agonists, KD3010 and T3D-959, caused a significant increase in *Ucp2* expression levels. (**B**) The TDP-43^Q331K^ model was chosen to represent the drug target engagement testing for KD3010. Results are shown for WT (open column), glycine-vehicle (green column), or KD3010 treatment (red column). There was no significant elevation in *Ucp2* or *Angptl4* expression levels after 10 months of drug treatment. (**C**) The TDP-43^Q331K^ model was again chosen to test drug–target engagement. Results are shown for WT (open column), saline-vehicle (green column), and T3D-959 treatment (red column). There was a significant elevation in both *Ucp2* and *Angptl4* expression after 10 months of drug treatment. *p* < 0.05, * and *p* < 0.01, ** one-way ANOVA with Tukey’s post hoc test. Error bars represent ±SD.

Target engagement by the two PPARδ agonists was assessed following chronic dosing over the 9–10-month treatment period. As a reference for target engagement, three-month-old WT mice not included in the longitudinal study were treated by intraperitoneal (IP) injection with KD3010 (30 mg/kg) or T3D-959 (50 mg/kg) for five consecutive days. Brain and spinal cord tissue were subsequently collected for RNA isolation and RT-PCR analysis. Expression of two genes known to be upregulated by PPARδ activation [28], *uncoupling protein 2 (Ucp2)* and *angiopoietin-like 4 (Angptl4)*, was examined. Both genes showed robust upregulation in the cortex following acute treatment, with *Ucp2* expression increased by 83% after KD3010 treatment and by 146% after T3D-959 treatment (Figure 9A). Following completion of the chronic dosing regimen, cortical tissue from TDP-43^Q331K^ mice treated with KD3010 was analyzed to assess sustained target engagement. In these mice, neither *Ucp2* nor *Angptl4* expression was elevated relative to controls (Figure 9B). In contrast, TDP-43^Q331K^ mice treated with T3D-959 exhibited strong upregulation of both target genes, with approximately 200% and 500% increases in *Ucp2* and *Angptl4* expression, respectively, relative to WT controls (Figure 9C).

## 3. Discussion

### 3.1. Behavioral Analysis

This preclinical study evaluated the effects of two PPARδ agonists in two extensively characterized ALS/FTD mouse models, with treatment initiated prior to or near the onset of reported disease phenotypes and maintained over a prolonged duration. This design was intended to maximize the likelihood of detecting therapeutic benefit across behavioral domains relevant to ALS and FTD. Overall, behavioral outcomes differed substantially between the two models. The AAV-mediated C9-149R model exhibited only mild and inconsistent neurological phenotypes across multiple assays, consistent with previous reports describing relatively subtle motor involvement in this system. In contrast, the TDP-43Q331K transgenic model developed robust, progressive motor and behavioral deficits, providing a clear opportunity to assess therapeutic efficacy. Neither KD3010 nor T3D-959 produced consistent improvements in body weight, motor coordination, grip strength, neurological composite score, contextual fear conditioning, or open field performance in either model. In the TDP-43^Q331K^ mice, where deficits were pronounced and progressive, both drugs failed to mitigate motor or behavioral decline despite long-term dosing. In the C9-149R model, the limited severity and variability of behavioral phenotypes constrained interpretation of potential rescue effects.

An unexpected but consistent finding across assays was the presence of baseline differences between WT and C9-2R control mice, including reduced body weight and altered freezing behavior. These effects were observed independently of the pathogenic 149R repeat expansion and suggest that AAV-mediated C9 expression itself may influence metabolic or behavioral outcomes. While the mechanisms underlying these differences remain unclear, their reproducibility across multiple endpoints highlights an important consideration for interpretation of AAV-based C9 models. Despite these model-specific complexities, the overall behavioral findings converge on a consistent conclusion: pharmacologic activation of PPARδ does not confer measurable functional benefit in either ALS/FTD mouse model under the conditions tested. This lack of efficacy was observed even in the context of sustained target engagement for T3D-959, indicating that failure to modify disease phenotypes cannot be attributed solely to inadequate drug exposure or pathway activation.

### 3.2. Neuroanatomy Analysis

Across both ALS/FTD mouse models examined, we did not observe significant loss of choline-acetyltransferase-positive (ChAT^+^) motor neurons or consistent neuromuscular junction (NMJ) pathology at one year of age. Although an early characterization of the TDP-43^Q331K^ model reported a mild reduction in ChAT^+^ motor neurons [18], subsequent studies have emphasized functional and molecular abnormalities rather than overt motor neuron loss as defining features of this model. The absence of robust ChAT^+^ motor neuron loss or NMJ disruption in the present study does not preclude the presence of significant disease-related dysfunction, as multiple ALS mouse models exhibit pronounced behavioral and molecular phenotypes in the absence of extensive motor neuron degeneration [29,30]. Instead, these findings suggest that, under the conditions tested, neither model progresses to end-stage neuroanatomical degeneration within the examined timeframe and that functional impairments likely arise from neuronal dysfunction rather than pronounced cell loss. Consistent with this interpretation, the lack of a measurable neuroanatomical phenotype limited the ability to assess potential neuroprotective effects of KD3010 or T3D-959 at the level of motor neuron survival or NMJ integrity.

### 3.3. Neurofilament Light Chain: A Biomarker of Neuronal Health

NfL provided a sensitive and objective biomarker of neuronal injury across both ALS/FTD models examined. In the AAV C9-149R model, NfL levels were modestly elevated relative to C9-2R controls at one year of age, consistent with previous reports [27]. In contrast, the TDP-43^Q331K^ model exhibited markedly higher NfL levels compared to WT mice, indicating substantial neuronal dysfunction and aligning with the more aggressive behavioral phenotype observed in this model. Importantly, pharmacologic activation of PPARδ with either KD3010 or T3D-959 did not reduce plasma NfL levels in either model. This absence of effect was observed despite prolonged treatment duration and, in the case of T3D-959, sustained target engagement. These findings indicate that PPARδ agonism does not mitigate ongoing neuronal injury as measured by this robust translational biomarker.

### 3.4. PPARδ Agonists KD3010 and T3D-959 Fail to Rescue ALS/FTD Mouse Models

Across two ALS/FTD mouse models with markedly different disease severity, chronic pharmacologic activation of PPARδ failed to produce measurable therapeutic benefit. In the AAV C9-149R model, treatment was initiated at a time point corresponding to the earliest reported emergence of disease-associated molecular and behavioral features. Although several assays revealed subtle and occasionally significant phenotypes, neither KD3010 nor T3D-959 produced consistent or reproducible rescue effects across behavioral, biomarker, or molecular endpoints. In contrast, the TDP-43^Q331K^ transgenic model developed robust and progressive motor and neurological deficits, providing a stringent context in which to assess therapeutic efficacy. Despite early treatment initiation and the presence of multiple, well-defined disease phenotypes, neither PPARδ agonist mitigated functional decline, biomarker elevation, or molecular pathology. Thus, across both mild and aggressive disease contexts, PPARδ activation was insufficient to modify ALS/FTD-relevant outcomes.

Importantly, the lack of efficacy cannot be attributed solely to inadequate drug exposure or pathway activation. T3D-959 demonstrated sustained target engagement following chronic dosing yet failed to alter disease phenotypes. In contrast, KD3010 exhibited loss of target engagement consistent with the development of pharmacologic tolerance, a known limitation of some nuclear receptor agonists. Potential mechanisms contributing to reduced responsiveness include receptor downregulation, activation of compensatory metabolic or inflammatory pathways, epigenetic remodeling of target gene regulation, and crosstalk with other nuclear receptors [31,32,33,34]. Taken together, these findings indicate that activation of PPARδ alone is insufficient to confer disease-modifying benefit in ALS/FTD mouse models even under conditions of sustained target engagement. This underscores the need to reconsider the therapeutic relevance of metabolic pathway modulation in ALS and highlights the importance of rigorous preclinical evaluation across multiple models and endpoints.

Taken together, the findings across behavioral, neuropathological, biomarker, and molecular analyses converge on a consistent conclusion: pharmacologic activation of PPARδ does not confer disease-modifying benefit in these ALS/FTD mouse models. This lack of efficacy was observed across two mechanistically distinct disease contexts, including a model with relatively mild and variable phenotypes (C9-149R) and a model with robust, progressive motor and behavioral decline (TDP-43^Q331K^). Importantly, failure to observe therapeutic benefit cannot be attributed solely to inadequate target engagement, as sustained induction of PPARδ-responsive genes was confirmed following chronic T3D-959 treatment. Rather, these data suggest that modulation of metabolic and mitochondrial pathways downstream of PPARδ is insufficient, on its own, to alter disease-relevant outcomes in ALS/FTD models. The absence of molecular or functional rescue, even under conditions of prolonged treatment and confirmed pathway activation, represents a central and informative finding of this study and underscores the value of publishing well-powered negative preclinical data to guide therapeutic prioritization.

From a molecular perspective, the novelty of this study lies in demonstrating that sustained activation of a metabolically active nuclear receptor pathway, including robust induction of canonical PPARδ target genes, is insufficient to alter disease-relevant molecular, biomarker, or functional endpoints in ALS/FTD models. These findings argue against a simple causal link between enhancement of mitochondrial/metabolic programs and disease modification in ALS and instead suggest that such pathways may be permissive or compensatory rather than disease-driving. By defining the molecular limits of PPARδ agonism in vivo, this work provides a mechanistically informative boundary for future therapeutic strategies

## 4. Materials and Methods

### 4.1. Animal Studies and Preclinical Trials

All animal experiments complied with National Institutes of Health (NIH) regulations and UCI’s IACUC guidelines. After genotyping, mice were weighed, and littermates were assigned to balanced groups based on weight and gender. Mice received intraperitoneal injections of T3D-959 (50 mg/kg per day in saline) or KD3010 (30 mg/kg per day in 150 mM glycine buffer, pH 8.5) on a Monday–Wednesday–Friday schedule. Treatment began at 3 months of age for C9-AAV mice and 2 months for TDP-43^Q331K^ mice. Blinded observers conducted regular behavioral testing. Mice were euthanized after one year, and histopathology analyses were blinded also. The TDP-43^Q331K^ transgenic mouse line was originally generated and characterized as described previously [18] and has been widely used in ALS research. Mice were maintained on a C57BL/6 background. The AAV-mediated C9-149R model was generated by intracerebral delivery of AAV encoding 149 G4C2 repeats, as previously described and characterized [15,27]. Transgenic mice were genotyped using established PCR-based methods as previously described [18]. Mice were housed in a temperature- and humidity-controlled facility on a 12 h light/dark cycle with ad libitum access to food and water.

### 4.2. Behavioral Tests

Cohorts of sex- and age-matched test condition animals, along with littermate controls, were evaluated using a range of behavioral tests, including the accelerating rotarod, grip strength, neurological assessment, contextual fear conditioning, and open field assay. All behavioral equipment was sanitized with 30% ethanol before and between each use. After completing each test, mice were returned to their home cages. All behavioral assessments, including the composite neurological score, were conducted by observers blinded to genotype and treatment condition.

#### 4.2.1. Rotarod Assessment

Mice were assessed for time to fall on an accelerating rotarod treadmill (ENV-574M, Med Associates, Inc., St. Albans, VT, USA) set to increase from 4 to 40 rpm over four trials, with a maximum duration of 300 s per trial. Testing occurred at specific intervals: 2, 4, 6, 8, 10, and 12 months of age for TDP-43^Q331K^ mice and 3, 6, 9, and 12 months for C9-AAV mice. A 5 min rest period was provided between trials. Latency to fall, measured in seconds, was recorded when mice either fell or rotated three times around the bar. Following three days of training (three trials/day), data from the fourth day was used for analysis.

#### 4.2.2. Grip Strength

Mice were assessed for forelimb and all-limb grip strength using a grip meter (Bioseb #BIO-GS3, Grip Strength Meter, Bioseb, Vitrolles, France) at each time point (2, 4, 6, 8, 10, and 12 months for TDP-43^Q331K^ mice; 3, 6, 9, and 12 months for C9-AAV mice). Each mouse was held by the tail and lowered toward the grip grid until it held on with two or all four paws. Gentle pulling was applied until the mouse was released. This procedure was repeated four times, and the data presented represent the average of the highest force recorded across trials.

#### 4.2.3. Composite Neurological Score

Mice were evaluated for neurobehavioral abnormalities at each time point (2, 4, 6, 8, 10, and 12 months for TDP-43^Q331K^ mice; 3, 6, 9, and 12 months for C9-AAV mice) using a previously described protocol [25]. Pathology was graded on a four-point scale (0–3, with 0 indicating no pathology and 3 the most severe) across four tests for a maximum combined score of 12: hind-limb clasping, gait, kyphosis, and ledge tests. The presented data represent the composite score for each animal across all four tests. As the resulting scores tended to be discontinuous, we employed a box and whiskers plot to display the data. The median is denoted by a black line with the colored box representing the middle 50% of data, and the whiskers are the minimum and maximum data points.

#### 4.2.4. Contextual Fear & Conditioning

Mice were tested for freezing behavior using a chamber with a grid floor capable of delivering mild electric shocks (Coulbourn Instruments). Each mouse was placed in the chamber for 2 min before exposure to an 80 dB white noise (conditioned stimulus, CS) for 30 s, followed by a mild foot shock (2 s, 0.5 mA; unconditioned stimulus, US). This CS-US pairing was repeated after 2 min. Mice were removed 30 s later and returned to their home cages. The next day, each mouse was reintroduced to the chamber, and freezing behavior was recorded for 5 min without a CS–US pairing. Freezing was captured using a mounted camera and analyzed with FreezeFrame 4 software (Coulbourn Instruments, Whitehall, PA, USA). TDP-43^Q331K^ and C9-AAV mice were tested at 12 months of age. Data represents the percentage of time spent freezing during the second-day trial without a CS-US pairing.

#### 4.2.5. Open Field

Mice were placed in the center of an open field apparatus (SuperFlex Open Field, Omnitech Electronics, Inc., Omnitech Electronics, Columbus, OH, USA) and allowed to explore the chamber freely for 15 min. Movement was recorded and analyzed using Fusion Software (Omnitech Electronics, Inc., version 6.3, Omnitech Electronics, Columbus, OH, USA). TDP-43^Q331K^ and C9-AAV mice were tested after 6 months of treatment and again at 12 months of age. Data represents the total distance traveled by each mouse during the exploration period.

### 4.3. Immunohistochemistry

Euthanized mice underwent transcardial perfusion with 4% paraformaldehyde. The spinal cord was cryoprotected in 30% sucrose and the quadriceps in 20% sucrose. Tissues were embedded in TissueTek O.C.T. (Sakura Finetek, Torrance, CA, USA), and 30 µm sections were prepared: cross-sections from the lumbar spinal cord and longitudinal sections from the quadriceps. Lumbar spinal cord sections were immunostained with antibodies against ChAT (1:100, Sigma-Aldrich, St. Louis, MO, USA), Neurotrace (1:1000, Invitrogen, Carlsbad, CA, USA), and Hoechst (1:10,000, Invitrogen, Carlsbad, CA, USA). Quadriceps sections were immunostained with Synaptophysin (1:250, Invitrogen, Carlsbad, CA, USA), Alpha-Bungarotoxin (1:1000, Invitrogen, Carlsbad, CA, USA), and Neurofilament-L (1:1000, Cell Signaling Technology, Danvers, MA, USA). Secondary antibodies (1:5000, Invitrogen™) were used for detection, and sections were mounted with ProLong™ Gold antifade media (Invitrogen, Carlsbad, CA, USA).

### 4.4. Quantification of ChAT^+^ Motor Neurons

Images were captured using an Echo Revolution microscope (Echo Laboratories, San Diego, CA, USA). ChAT+ motor neurons in the ventral horn were counted from at least 10 ventral horns per animal at 12 months of age, using a minimum of three animals per genotype. The data presented represents the average number of ChAT+ motor neurons in the ventral horn.

### 4.5. Quantification of NMJs

A minimum of 10 images were taken with the Nikon A1RHD25 resonant scanning confocal (Nikon Instruments Inc., Melville, NY, USA), and a minimum of 20 NMJs in the gastrocnemius were counted from four animals per genotype at 12 months of age. The data shown is the percentage of innervated, partially denervated, and fully denervated NMJs from each animal.

### 4.6. Quantification of pTDP-43 and Poly(GP) Repeats

Euthanized mice were transcardially perfused with 4% paraformaldehyde, and brain tissues were also submerged fixed in 4% paraformaldehyde. Hemi-brains were then embedded in paraffin. Immunohistochemistry for DPR protein inclusions and pTDP-43 were performed as previously described in [15].

### 4.7. Detection of Neurofilament Lite Chain in Plasma

Blood samples were collected from the right ventricle and placed into K2 EDTA tubes (BD Microtainer^®^ tube., Becton, Dickinson and Company (BD), Franklin Lakes, NJ, USA). The samples were centrifuged at 2000× *g* for 10 min at 4 °C to obtain plasma. Plasma NfL concentrations were determined using Simoa^®^ NF-Light Advantage Assay Kit (Quanterix Corporation, Billerica, MA, USA) and run on the HD-X Analyzer (Quanterix Corporation, Billerica, MA, USA).

### 4.8. RT-PCR

RT-PCR was performed on cDNA synthesized from the cortex and spinal cords of mice using Superscript™ IV VILO™ Master Mix (Invitrogen, Carlsbad, CA, USA). To analyze candidate splicing targets, 5 ng of cDNA was amplified for 26 cycles for Eif4h and Kcnip2 and loaded onto a 2% agarose gel. Gel images were captured, and isoform quantification was done using Bio-Rad Chemidoc (Bio-Rad Laboratories, Hercules, CA, USA) and ImageLab software (Bio-Rad Laboratories, version 6.1, Hercules, CA, USA). The intensity ratios between products, including the cassette exon and exon skipping, were averaged from at least three biological replicates per genotype.

## 5. Conclusions

Using two well-characterized ALS/FTD mouse models and a comprehensive behavioral, pathological, and molecular assessment strategy, we found no evidence that pharmacologic activation of PPARδ confers therapeutic benefit. Neither KD3010 nor T3D-959 rescued disease-associated phenotypes in the AAV C9-149R model or mitigated the robust motor deficits observed in the TDP-43^Q331K^ model even under conditions of sustained target engagement. Furthermore, baseline differences observed between AAV C9-2R and WT control mice across select behavioral assays underscore the importance of carefully interpreting outcomes in AAV-based C9orf72 models. Collectively, these findings indicate that PPARδ agonism alone is insufficient to modify ALS/FTD-relevant phenotypes and highlight the necessity of rigorous, multi-model preclinical evaluation when prioritizing therapeutic strategies for ALS and related neurodegenerative disorders.

## Data Availability

The datasets generated and analyzed during the current study are available from the corresponding authors upon reasonable request.

## Supplementary Materials

The following supporting information can be downloaded at: https://www.mdpi.com/article/10.3390/ijms27041820/s1.
