# Supplementary figures and images for: PPAR-Delta Agonist Therapies Did Not Rescue Hallmark Disease Phenotypes in Two Sets of Preclinical Trials in ALS TDP-43 and C9orf72 Model Mice
Source: Int J Mol Sci. 2026 Feb 13;27(4):1820. doi: 10.3390/ijms27041820 (PMC12940718; doi:10.3390/ijms27041820)

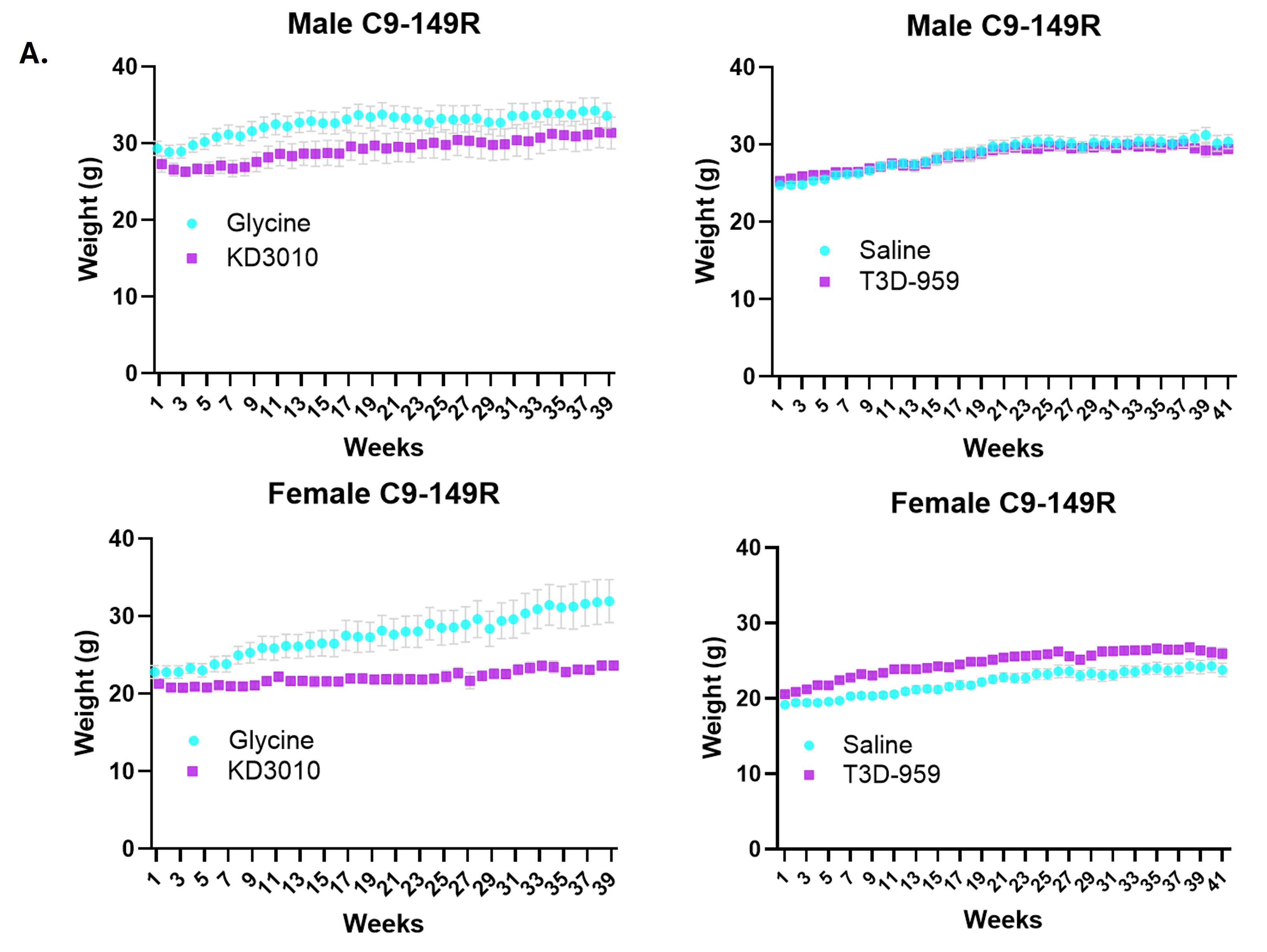

Supplement: Supplementary file 1 [file ijms-27-01820-s001.zip › Suppl. Fig. S1A.jpg]

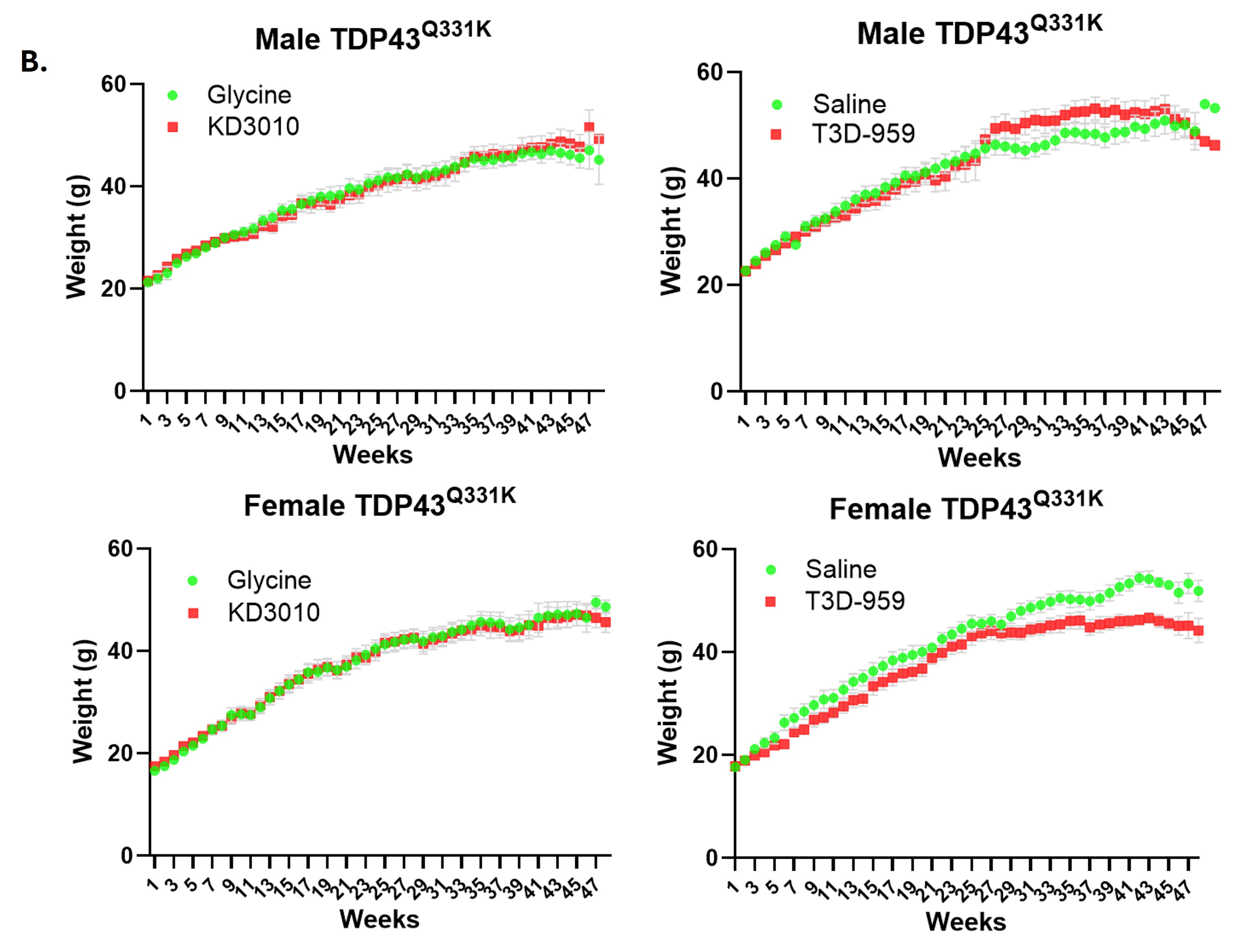

Supplement: Supplementary file 1 [file ijms-27-01820-s001.zip › Suppl. Fig. S1B.jpg]

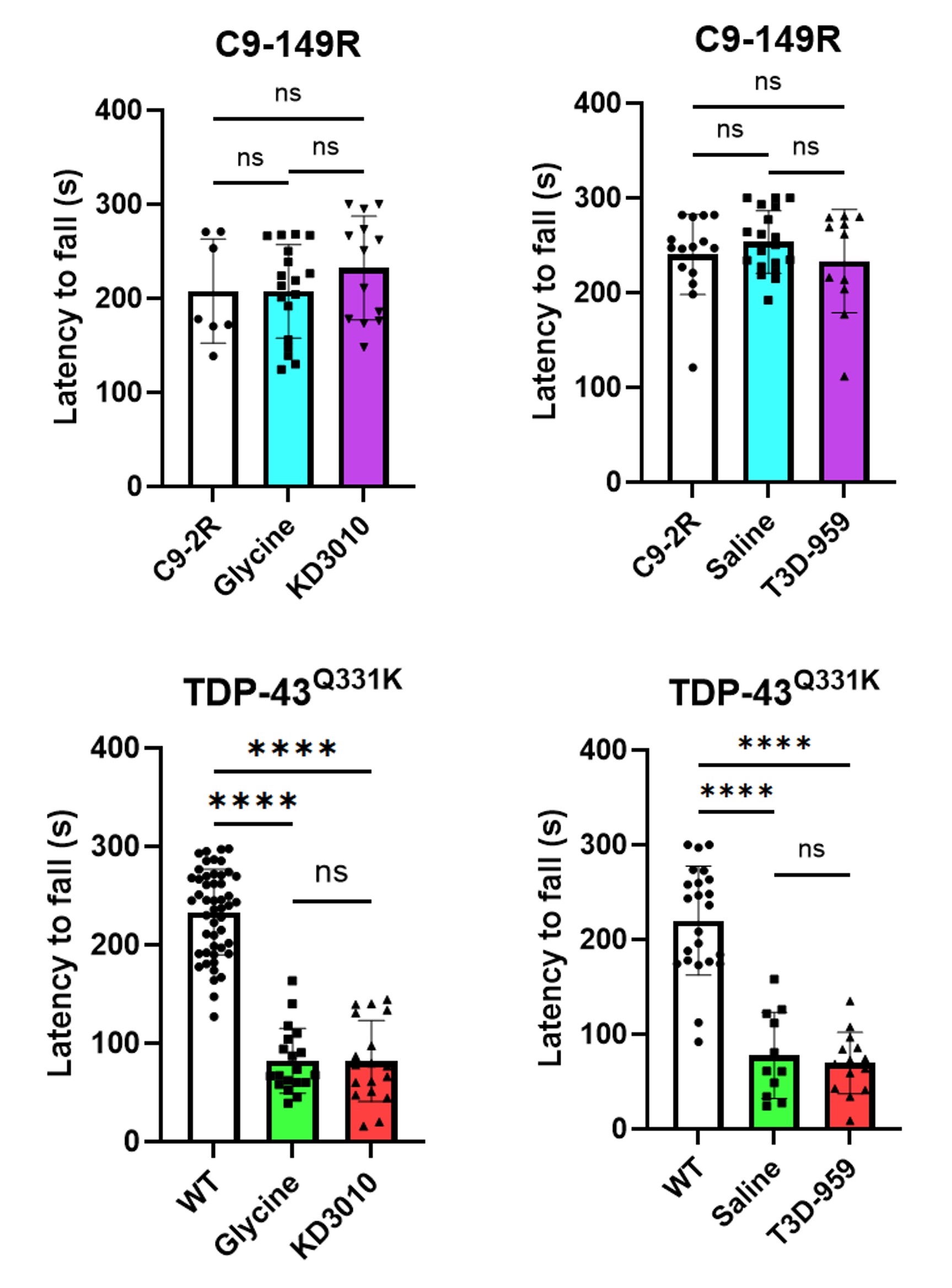

Supplement: Supplementary file 1 [file ijms-27-01820-s001.zip › Suppl. Fig. S2.jpg]

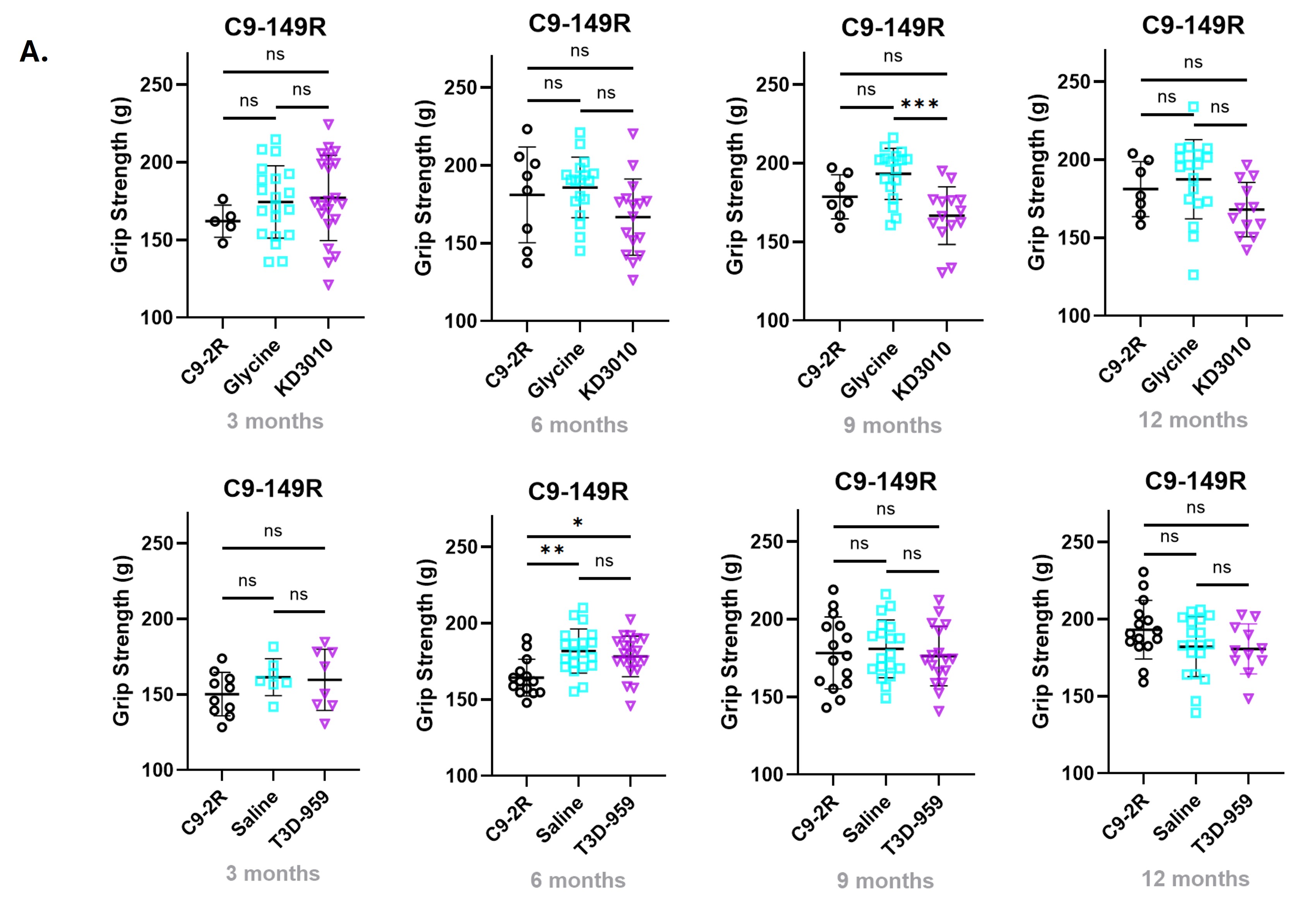

Supplement: Supplementary file 1 [file ijms-27-01820-s001.zip › Suppl. Fig. S3A.jpg]

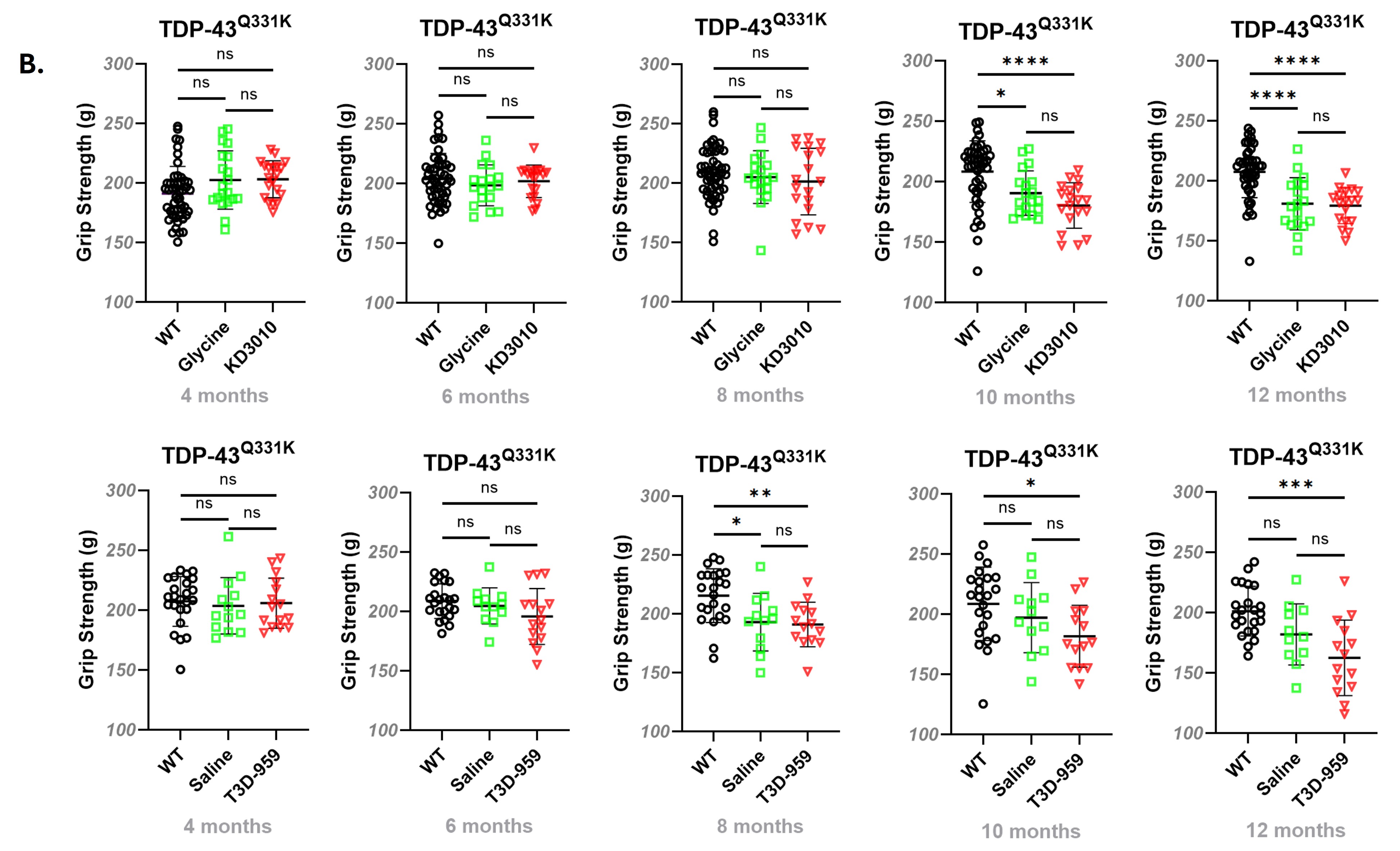

Supplement: Supplementary file 1 [file ijms-27-01820-s001.zip › Suppl. Fig. S3B.jpg]

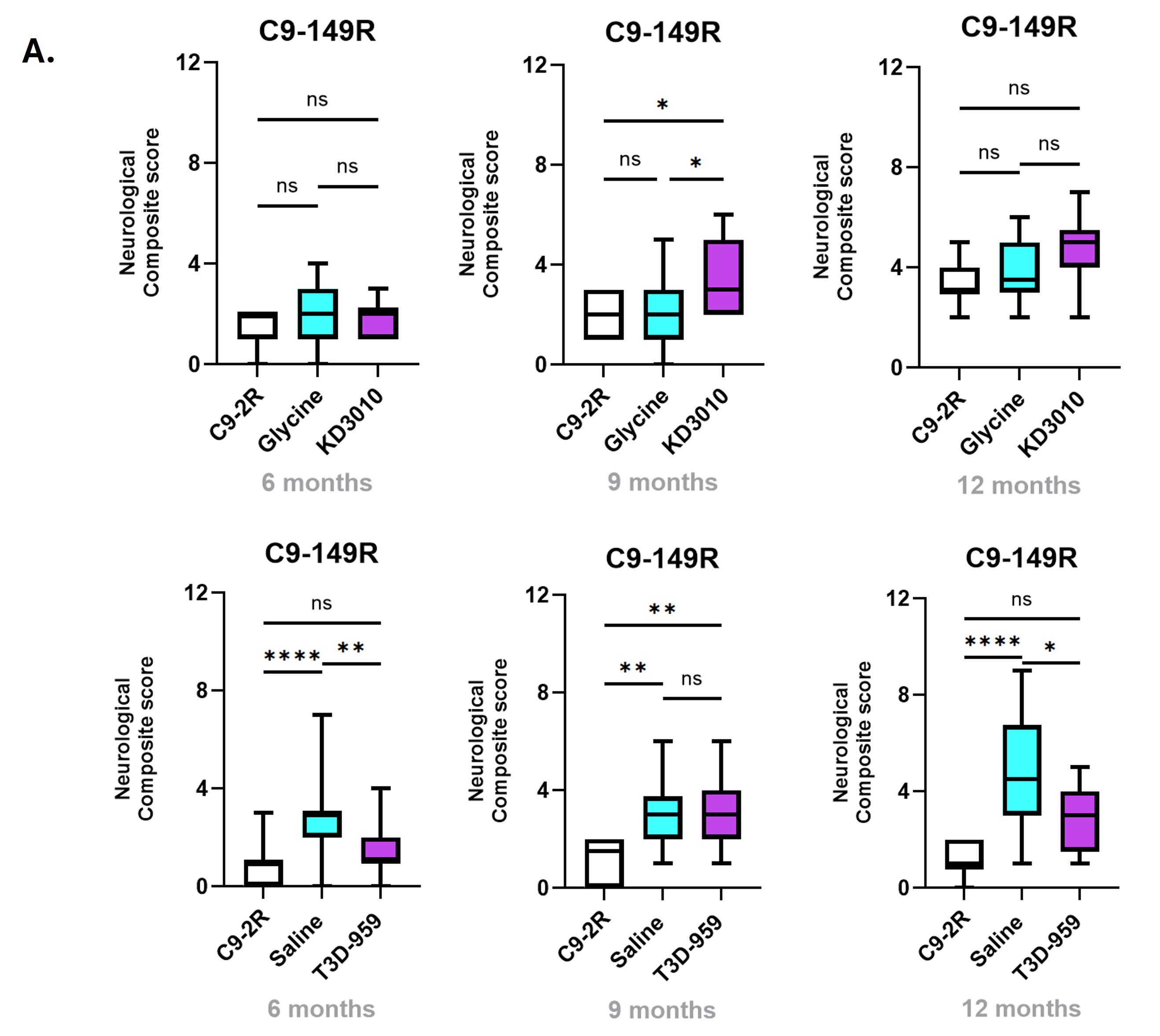

Supplement: Supplementary file 1 [file ijms-27-01820-s001.zip › Suppl. Fig. S4A.jpg]

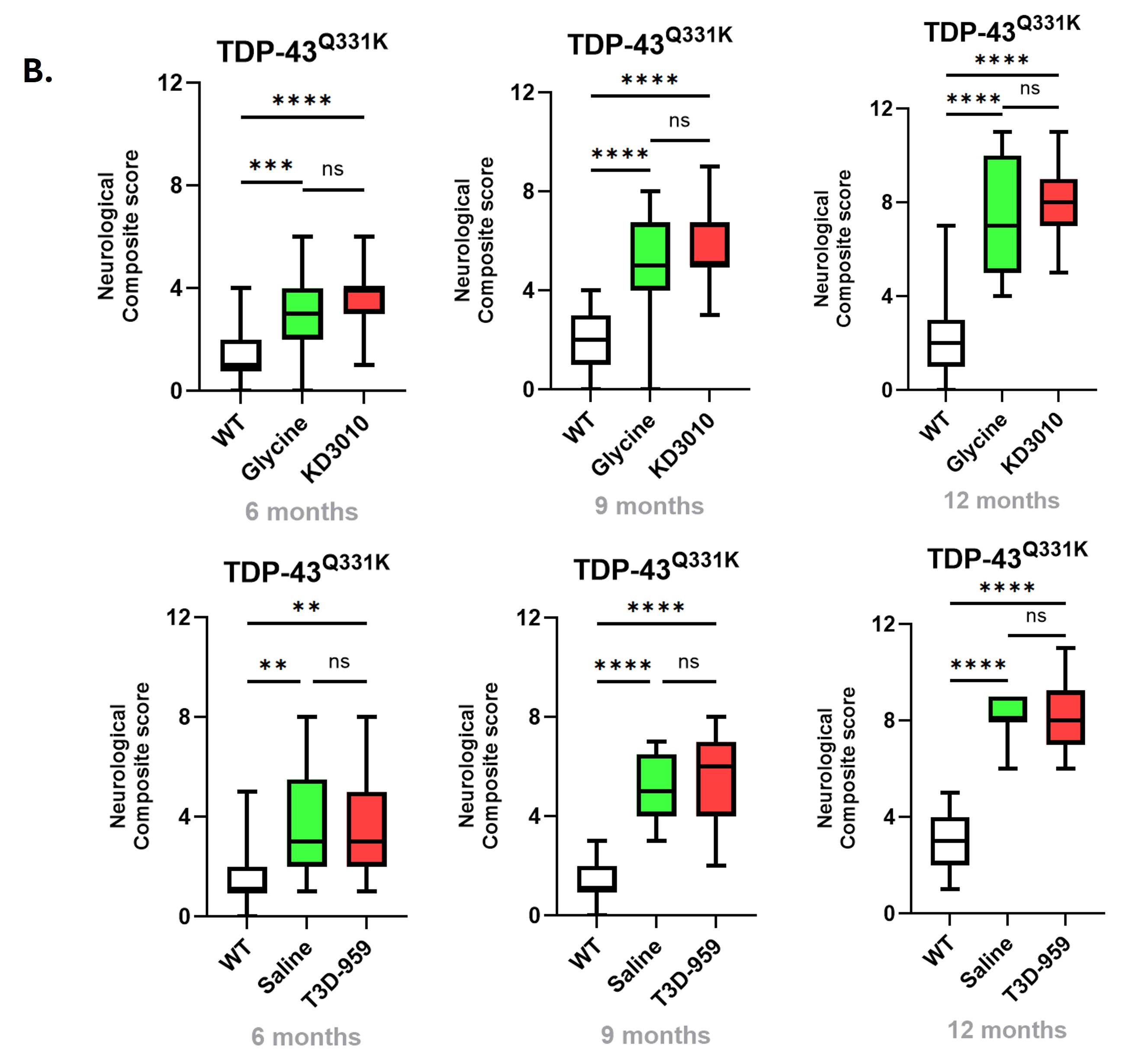

Supplement: Supplementary file 1 [file ijms-27-01820-s001.zip › Suppl. Fig. S4B.jpg]

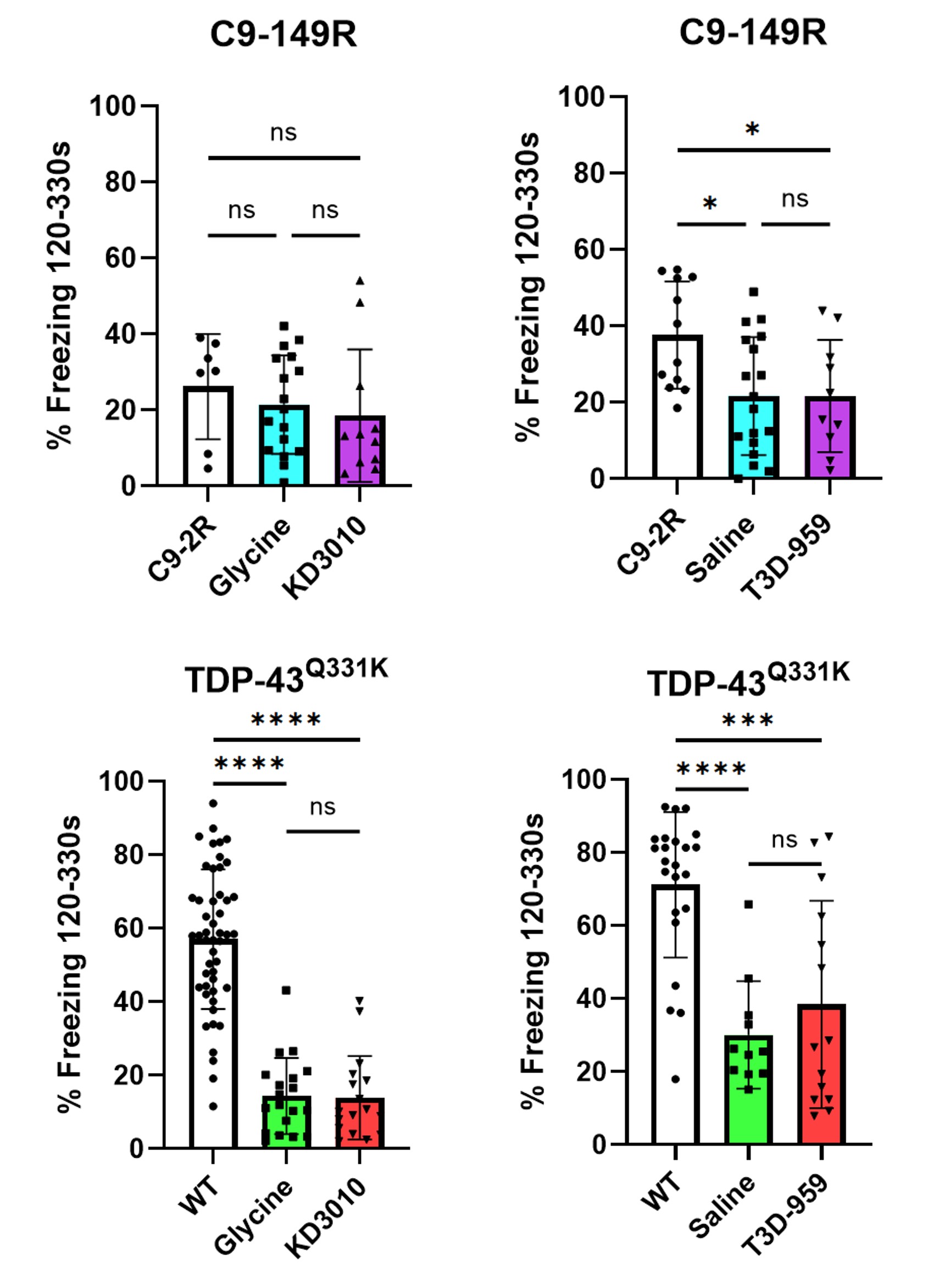

Supplement: Supplementary file 1 [file ijms-27-01820-s001.zip › Suppl. Fig. S5.jpg]
